# The Costs of Living Side-by-Side with Monkeys: Economic Impacts on Commercial Farms and Property by Toque Macaques and Proposed Deterrent Strategies in a Rural Agriculture Area of Kurunegala District, Sri Lanka

**DOI:** 10.3390/ani15030366

**Published:** 2025-01-27

**Authors:** S. D. Yeshanthika Jayarathne, Charmalie A. D. Nahallage, Michael A. Huffman

**Affiliations:** 1 Department of Anthropology, University of Sri Jayewardenepura, Nugegoda 10250, Sri Lanka; yeshani.jayarathna@gmail.com (S.D.Y.J.); chamalie@sjp.ac.lk (C.A.D.N.); 2 International Center for Multidisciplinary Studies, University of Sri Jayewardenepura, Nugegoda 10250, Sri Lanka; 3 Institute of Tropical Medicine, Nagasaki University, Nagasaki 852-8521, Japan

**Keywords:** agricultural and property damage, endemic species, human–wildlife conflict, *Macaca sinica sinica*

## Abstract

As the human population has grown and expanded, increasing pressure is being put on natural habitats in Sri Lanka. This situation has led to a noticeable increase in human–primate conflicts. To understand the situation, we studied the interactions between humans and macaques in three administrative divisions of the Kurunegala District. Data was gathered from 875 informants through interviewer-administered questionnaires between 2020 and 2022. The monthly economic loss by commercial farmers due to macaque damage to fruits and vegetables doubled by 2022, amounting to approximately 5000 LKR. In non-fruiting seasons, losses from coconut damage increased, ranging from 3000 to 14,000 LKR/month, decreasing by over 50% during fruiting seasons. Property damage per household averaged between 850~4000 LKR/month. A cost of approximately 1200~3000 LKR was borne per household/month to deter monkeys from the fields. Macaques were the primary culprits for crop damage in this area, and were also responsible for property damage, surpassing that of other animals. The consensus among the community is that either relocating macaques to other forested areas or sterilizing them to control their population could mitigate the issue to some extent. An integrated management plan involving relevant stakeholders including the Forest Department, the Wildlife Conservation Department, the local agricultural agency, and local citizens is necessary to address the conflict arising from human–macaque crop utilization.

## Simple Summary

An integrated management plan is recommended to address these conflicts effectively. In Sri Lanka, conflicts between humans and primates, particularly macaques, have increased due to habitat loss and human population growth. A study conducted with an interviewer-administered questionnaire and field observations in the Kurunegala District revealed significant economic losses to farmers from crop and property damage caused by macaques. Monthly losses ranged from 2300 LKR to 14,000 LKR, depending on the crop and season, with additional costs of 1200 LKR to 3000 LKR per household for deterrent methods like firecrackers and air rifles.

## 1. Introduction

Human–primate conflict in Sri Lanka predominantly involves three diurnal primate species: the Toque Macaques (*Macaca sinica*), Tufted Grey Langur (*Semnopithecus priam thersites*), and Purple-faced Langur (*Semnopithecus vetulus*). Conversely, the two nocturnal *Loris* spp. have minimal interactions with humans and are not associated with crop or property damage, thus there is no conflict with them. Macaques, known for their sociable nature, frequently interact with humans and are often found near human settlements. In contrast, langurs generally prefer natural environments and natural diets, exhibiting less inclination towards human interaction [1,2,3]. Purple-faced leaf langurs are typically arboreal folivores with minimal human interactions, although this may vary regionally [2,3,4,5]. Toque Macaques, too, exhibit dietary preferences influenced by habitat; those in natural environments primarily consume plant material, while those in urban or temple areas often rely on food provided by pilgrims, particularly leftovers from temple offerings [1,2,6].

The history of conflict between humans and primates in Sri Lanka is well-documented. Robert Knox, an English traveler who spent approximately two decades living freely in various regions of the country under the terms of his imprisonment by the Kandyan King, recorded instances of macaques invading corn fields and home gardens despite concerted human efforts to guard them [7]. Additionally, folk poems have also portrayed incidents of crop raiding by primates [8]. Presently, crop raiding is widespread across all 25 districts of Sri Lanka, influenced by factors common to other countries, such as primate species, types of crops, seasonal variation, proximity of villages to forests, availability of natural food sources, and methods employed by humans to guard crops [9,10]. Except for the nocturnal *Loris* spp., all primates are considered pests to varying degrees in the provinces where they are found [2,3,6,11,12,13]. In areas where all three diurnal primates coexist, toque macaques generally cause the most crop damage, followed by gray langurs [6]. However, in certain regions of the North Central province, gray langurs inflict more damage than toque macaques [14].

Crop damage inflicted by animals poses a significant challenge for small-scale subsistence farmers, especially in countries like Sri Lanka, where around 28.5% of the population depends on agriculture [15]. Various animal species contribute to agricultural losses through foraging on crops [16]. Besides parasitic invertebrates, birds [17,18], rodents [19,20], mouse deer [21], porcupines [21], wild boar [22,23], and elephants [24,25,26], non-human primates [2,27] are recognized as pests causing negative interactions between humans and wildlife. Among these, non-human primates are globally acknowledged as the most destructive crop raiders [10,12,28,29,30,31]. Primates belonging to the genera *Macaca*, *Papio*, and *Cercopithecus* are frequently mentioned as problematic pest species [16]. Their social organization, cooperative behaviors, communication abilities, intelligence, dietary and behavioral flexibility, manual dexterity, and agility collectively present significant challenges for farmers endeavoring to safeguard their crops [16]. The interaction between humans and monkeys in Sri Lanka has intensified in recent decades due to various factors, including agricultural, irrigation, and industrial developments, as well as urban expansion and fragmentation of natural forest habitats driven by human population growth [18,31]. Forest fragmentation, particularly in the wet and dry zones, has led to primates increasingly encroaching on farms and agricultural lands in search of food, heightening conflicts [6]. Hence, our study seeks to evaluate the extent of human–monkey conflict by documenting crop and property damage caused by *Macaca sinica sinica* (the only diurnal species present in the area and the sole primate source of crop damage) as well as assessing the resulting household losses in villages adjacent to the Balagalla reserved forest, Kurunegala District in the North Western Province of Sri Lanka. This site was selected because there have previously been no studies conducted, and the local authorities have not taken any substantial actions to address crop damage.

## 2. Materials and Methods

### 2.1. Study Area

**Figure 1:**
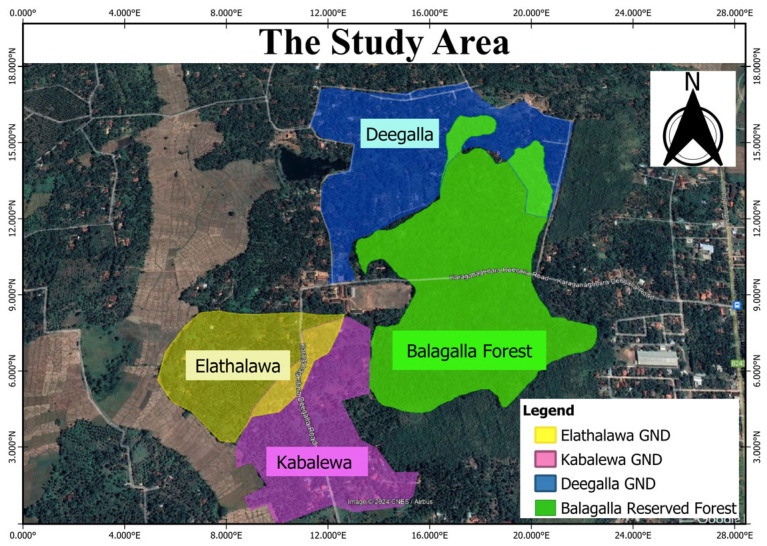
The study area.

Deegalla, Kabalewa, and Elathalawa Granaseva Niladari Divisions (GND) (7°28′19.6428″ N & 80°2′40.6392″ W) were selected based on initial observations indicating a higher intensity of interaction between humans and macaques compared to other GNDs (Figure 1) where we can find only Toque Macaques. The predominant habitat in the study area consisted of village home gardens featuring tall fruit tree species such as *Artocarpus heterophyllus* (Jack fruits) (56%), *Mangifera indica* (Mango) (61%), *Cocos nucifera* (Coconut) (93%), and *Areca* (Areca nut) (34%). Medium-sized fruit tree species, including *Nephelium lappaceum* (Rambutan) (59%), *Carica papaya* (Papaw) (48%), *Musa paradisiaca* (Banana) (34%), and *Psidium guajava* (Guava) (20%) were also prevalent. These three GNDs are linked to the “Balagalla Reserved Forest”, the sleeping area of all three groups of macaques, and a necessary refuge for them. Deegalla GND is located approximately 50 m from the forest, with the main road as the only boundary between Deegalla and the forest, which experiences low traffic. This area serves as a favored and proximal food source for macaques. Kabalewa GND is located approximately 100–150 m away, while Elathalawa GND is the farthest, situated approximately 150–200 m from the forest. Kabalewa lies between Deegalla and Elathalawa. The monthly average temperatures range between 19 °C and 30 °C, with monthly rainfall averaging from 152.21 mm to 200 mm.

### 2.2. Data Collection

Different sampling methods were used to select the sample size from the total number of families according to the situation of their category. In total, 875 samples were taken from two recognized categories (Table 1).

The Krejcie and Morgan Formula, commonly used in social sciences research, was used here for its simple and readily accessible method of calculating a sample size based on the total population size.

Three macaque troops were living in these GND, and their group age–sex compositions were verified (Deegalla: 27 (F), 10 (M); Kabalewa: 20 (F), 8 (M); and Elathalawa: 21 (F), 6 (M)) and observed using the focal animal sampling method [32].

The interviewer-administered questionnaire (*n* = 875) was conducted from July 2020 to June 2022 to gather data from farmers (*n* = 275) and villagers (*n* = 600) across three GNDs. All interviews were conducted in Sinhalese, the major language used by people in the area, with verbal consent obtained to ensure comprehensive information. The questionnaire was comprised of closed- and open-ended questions as well as binary/polar (yes/no) inquiries. Participants who had suffered crop damage were asked to provide detailed information regarding their losses during those years, including the types of crops damaged and the quantity lost each month. Additionally, they were inquired about their annual expenditures on measures to protect their crops, referred to as damage control costs. Further, they were asked about the alteration of farming practices of the use of land in response to macaque damage. 

Field observations amounted to 3705 h (1871 h in the coconut fields, 249 h 15 min in home gardens, and 1585 h within the villages), recording the property and crop damages at home gardens (fruits and vegetables) and coconuts. Data on the number of coconuts destroyed, the weight of fruits and vegetables destroyed in kilograms, the duration of the time macaques spent in the field, and weather conditions (raining/sunny) were collected daily. During field observations, we noted how many coconuts were destroyed, partially, and or detached from trees, preventing them from maturing to a sellable state. At the end of each day, we counted and weighed damaged fruits and vegetables. In the study area, coconut cultivation constituted a substantial source of revenue. We identified three primary coconut plantations to assess the financial impact of the predominant commercial crop. Coconut damage was calculated from a selected plantation from each GND, amounting in total to approximately 5.5. acres and 521 coconut trees. The total area of home gardens investigated was approximately 38 acres, with a variety of different vegetables and fruits grown.

At each home garden, kilograms of crops were taken to the local markets monthly, and the approximate weight of the crops damaged (kgs) was recorded. Coconut damage was quantified as the number of destroyed coconuts counted per day from each plantation. Prices for each crop were averaged over the period from 2020 to 2022 according to DCS & CBSL data. These average values were then applied to calculate the monetary losses for each crop per year. The total cost was determined by summing the values of actual damage caused by macaques and total expenditures on damage control measures. Consequently, for each year, information was gathered on crops regarding the value of actual macaque damage to crops, losses incurred from discontinuation of crop cultivation due to crop loss by macaques, and expenditures on damage prevention measures. The data obtained were presented as a percentage of respondents for each response.

## 3. Results

### 3.1. Demographic Information of the Respondents

The economic structure of households in the study area reflects a typical rural Sri Lankan lifestyle, with 16.9% of individuals being employed, while 83.1% are engaged in farming, with a small proportion also working as daily laborers. Despite their employment status, the majority of households are involved in cultivating fruit trees (*n* = 821) and crop plants (*n* = 791). All interviewed farmers were males, with ages ranging from 20 to 70 years.

Of the respondents, 21% had not received any formal education, while 79% had undergone some form of schooling. Among farmers, 43.8% have been engaged in farming throughout their adult lives, for an average duration of 12.1 years (SD = 6.7, range: 2 to 24 years), and 5.6% started farming after retiring from their previous jobs, with an average of 12.3 years of farming experience (SD = 6.5, range: 5 months to 21 years). The majority of the informants (81.3%) own the coconut fields and home gardens that they cultivate, with ownership periods ranging from 11 years to 4 generations. The household size of interviewees ranges from 1 to 6 people, with 0 to 4 dependents. The total income generated from the crops by each interviewee per month ranged between 10,000 LKR to 80,000 LKR.

Ten percent of the interviewees’ main monthly income came from their respective jobs, 74.4% from coconut fields and home gardens, 8.7% from home gardening, and 6.9% from their respective jobs and coconut fields.

### 3.2. Crop and Property Damage Caused by Macaques

#### 3.2.1. Types of Damage

Damage was classified into three categories: economic loss from damaged vegetables and fruits (Table 2), property damage (Table 3), and coconut damage (Table 4 and Table 5). The majority of the respondents experienced damage to fruits, leaves, and buds (98.2%), which are of commercial value. The preferred fruit species for macaques were banana, mango, and papaya (95%) (Table 2). Property damages primarily included damage to water taps and water sources (72.6%) and to roof tiles (68.2%). Such property damage resulted in an average loss ranging from 850 LKR to 4000 LKR per household per month.

#### 3.2.2. Seasonal and Diurnal Variation in Crop Damage

Respondents informed that the peak period of conflict was from July to September (rainy season), coinciding with the availability of fresh crops (vegetables and fruits), with 84.7% reporting this timeframe as the most problematic. In the dry season, the damage is reduced to 20%, however the cost is still considerable.

In the dry season (non-fruiting season), the damage to coconuts was higher because of the absence of other crops. During the day, mornings (6:00–11.00) and late afternoon to early evening (14:00–18.00) times were identified as the most critical periods for human–macaque conflict to occur. Conversely, night-time (18:00–6.00) was perceived as the period when conflict occurred the least. Out of 9053 observed macaque visits to home gardens, 5741 were successful, and 3312 were not successful. The success of raids was dependent upon whether people were at home or not. On average, these group home garden raids consisted of 32 individuals (ranging from 1 to 63 individuals). The frequency of macaque visits to the home garden remained more or less consistent throughout both non-fruiting and fruiting seasons, but the items eaten were different based on availability.

#### 3.2.3. Economic Loss Due to Crop Damage

Macaques were found to raid crops daily, resulting in an estimated loss of three kilograms of fruit per week, valued at approximately 1800 LKR, or 4% of the average monthly income), and five kilograms of vegetables per week from home gardens, valued at around 2000 LKR, or 4.44% of the average monthly income). Most home garden produce was sold at local markets to generate daily or weekly income for households.

More than 81% of the respondents stated that they had to purchase fruits from the market, despite growing them in their gardens, highlighting the impact of crop raiding on their expenses. Some vegetables, such as chilies, suffered damage from macaques traversing through home gardens.

A noticeable decrease in the number of trees and plants cultivated due to human–macaque conflict (HMC) (Table 2) was observed, resulting in increased household expenses. The majority of vegetable damage occurred due to the playful behavior of macaques and their careless roaming around croplands. Among the respondents, 89% reported that macaque damage led to either partial or complete abandonment of plants or cultivation, resulting in considerable economic loss. However, 11% of participants whose home gardens were not fully damaged managed to continue harvesting as before, albeit with less profit. They had to spend approximately 550 LKR to 5000 LKR to implement mitigation methods aimed at reducing crop damage caused by macaques (Table 6). Nevertheless, 94% of interviewees reported that expenditures for damage control were relatively minor compared to crop losses (Table 3). Water-related damages, such as water taps, are the most common, with a 72.6% incidence and costs ranging from 3500–4000 LKR. Roof damage (68.2%) and antenna damage (58.6%) are also prevalent, costing 1000–1500 LKR and 1500–2500 LKR, respectively. Items like kitchen essentials, telephone wires, and clothes experience moderate damage rates (30–50%), with costs varying based on the type of item. Overall, the data underscore the financial burden of recurring property damage, particularly for frequently affected areas like water systems and roofs.

Some specific crops like papaya experience higher losses due to macaques’ fruit food preference and the ease of processing and ingesting them.

We selected several fruits and vegetables that experienced significant crop damage from macaques to assess economic losses during the study period (Table 2). To quantify the economic loss per year, we utilized the monthly average price per kilogram of vegetables and fruits from the DCS and CBSL reports. The actual average monetary losses directly attributable to macaque activity increased annually, from over 2300 LKR in 2020 to approximately 5000 LKR by 2022.

#### 3.2.4. The Economic Loss Inflicted on Coconuts

Although the frequency of macaque visits to the coconut plantations remained more or less consistent throughout both the non-fruiting and fruiting seasons, the number of coconuts destroyed monthly varied (Table 4 and Table 5).

The fruiting season is the time of year that is warm enough for growth, especially for cultivated plants to grow. In this research, we consider the fruiting season to be synonymous with the home garden growing season. Thus, we were able to analyze the different attractants for macaques during the fruiting season in home gardens and non-fruiting seasons in the coconut fields.

This variation can be attributed to the abundance of alternative fruits and vegetables in the villages, which diverted the macaques’ attention away from the coconut plantations. We assigned a monthly selling price of 60 LKR per coconut to estimate economic losses. During the non-fruiting season, monthly economic losses ranged from 3000 LKR to 14,000 LKR, representing a visible decline of over 50% compared to the fruiting season, where losses ranged from 1200 LKR to 6000 LKR (Table 4 and Table 5).

#### 3.2.5. Economic Loss and the Distance to the Nearest Forest Reserve

Deegalla (30 m), the closest plantation to the forest, accounted for the highest number of coconuts destroyed during both seasons (105–220 coconuts destroyed per month in the non-fruiting season, 63–99 coconuts destroyed per month in the fruiting season), representing the highest economic losses (LKR 13,200 per month in non-fruiting season, LKR 5940 per month in fruiting season). Macaques visited this plantation more frequently (28 days/month in the non-fruiting season and 23 days/month in the fruiting season) than the other two plantations, which were located further away from the forest reserve, indicating that proximity to the forest increases the visits of macaques. The Kabalewa (150 m) and Elathalawa (200 m) plantations, which are farther away from the forest reserve, experienced comparatively low destruction and less economic loss. In Elathalawa, the farthest plantation from the forest reserve, the calculated economic loss was only 3600 LKR per month in the non-fruiting season and 1200 LKR per month in the fruiting season.

During the non-fruiting seasons, destruction and economic losses were higher across all plantations (Table 5). This is likely due to macaques seeking coconuts when other food sources are less abundant. Fruiting seasons recorded comparatively lower losses, and in Deegalla, losses dropped from LKR 13,200 to LKR 5940, indicating less damage when other food is available. When considering the relationship between plantation size and loss, it can be seen that larger plantations (e.g., Elathalawa with 3 acres and 200 trees) have lower per-tree destruction, suggesting that spread-out plantations may dilute the macaques’ impact on individual trees. Conversely, smaller plantations like Deegalla experience more concentrated damage, compounded by the fact that it was more easily accessible from the forest.

#### 3.2.6. Currently Used Deterrent Methods

The time needed for maintenance and the cost of materials appear to have been a strong consideration when deciding what methods to use to keep monkeys out of the plantations and fields (Table 6). The most commonly used deterrent methods (firecrackers and acrylic masks) incurred the lowest monthly costs on average.

## 4. Discussion

The phenomenon of wildlife foraging on human-grown crops has persisted since the advent of agriculture [33]. However, the severity of this issue is exacerbated in regions where human development encroaches upon previously untouched wildlife habitats [6]. Assessing the full extent of wildlife-related damage presents significant challenges [33]. Farmers often perceive losses to be greater than they are, especially when their plantations are near protected wildlife areas [34].

In our study, we estimated the annual economic losses incurred by commercial farmers due to macaques to be between 20,000 LKR and 25,000 LKR during the three years of our study. These figures likely underestimate the total damage caused by macaques to both human interests and agriculture, given this study’s specific focus. We only accounted for the economic impacts on commercial farmers caused by macaques. Moreover, our estimation method may have underestimated losses, as it did not consider multiple harvests per year for certain fruits and vegetables in the study area. If interviewees had abandoned susceptible crops, they could have potentially yielded multiple harvests, significantly increasing their total income. Thus, while our study highlights substantial economic impacts on agriculture due to macaque crop raids, it is imperative in the future to also assess damages caused by other wildlife species. The analyzed economic loss escalation in actual damage losses can largely be attributed to the expansion of the macaque population due to nutritionally better and more easily accessible human food resources and the absence of natural predators. Due to the ecological changes incurred with the human population expansion, development, agricultural projects, etc., there is a marked decrease in some animal populations, such as phytons, jackals, and birds of prey in the dry and intermediate zones of Sri Lanka. These species are known to be the natural predators of macaques. The decrease in the natural predators of macaques in Sri Lanka has contributed to the increase in macaque populations, leading to a range of ecological and human-related challenges. Without predators to regulate macaque numbers, their populations increase, and their behavior changes, resulting in more frequent and intense human–wildlife conflicts. Restoring or managing predator populations, along with effective human–wildlife coexistence strategies, could be another way to help address the negative impacts of macaque overpopulation in Sri Lanka.

The economic ramifications on agriculture in the study area are significant. Despite our conservative estimates, the identified losses are considerable and are likely to escalate even more with the proliferation of macaque populations in terms of both number and distribution area. It comes as no surprise that the majority of these losses were attributed to macaques. Given that this species favors tropical forest habitats, prevalent in the study area and adjacent to the croplands primarily under consideration, this correlation is not unexpected. However, we anticipate that as macaque populations expand into more remote GNDs, they may encounter increasingly more forest habitats, potentially deterring their advancement into farming areas. Nevertheless, should macaques extend their range into these areas, they are anticipated to become the primary cause of agricultural damage, particularly targeting fruit crops.

The main resting place of macaques was the Balagalla Reserved Forest, which is situated in the study area (Figure 1). The damage was proportional to the distance from the forest reserve to each coconut plantation (Table 4). The farthest plantation, Elathalawa, received considerably less damage than the plantation nearest to the forest. When considering the impact of macaques on crop damage, the distance between their habitat and the plantations plays a crucial role. The nearest plantation had a higher frequency of raids. Plantations closer to macaque habitats were more accessible, leading to more frequent raids by the macaques. The shorter distance means that macaques can expend less energy to reach the crops, making these plantations more attractive targets. With the ease of access and higher frequency of visits, the nearest plantations often suffered extensive crop damage. Macaques sometimes visit these areas multiple times a day, leading to continuous damage over the growing season. Macaques that frequently raid plantations may become more accustomed to human presence, leading to bolder behaviors and potentially more aggressive crop raiding.

Plantations farther from macaque habitats are less likely to be raided as frequently. The greater distance requires more energy and time for macaques to travel, making these plantations less appealing, especially if closer food sources are available. Due to the low frequency of visits to these plantations, they often experienced less crop damage overall. The damage may be more sporadic, with macaques only visiting these distant plantations when food is scarce in closer locations. When macaques did visit more distant plantations, they mostly targeted attractive crops, which were also high-price value cash crops, which could still result in significant but less frequent damage.

Presently, communities are grappling with various challenges as primates increasingly encroach upon home gardens in search of food [1,3,11,12,13,14]. Most gardens in the study area are modest in size, typically less than an acre, and suffer extensive damage from macaques, particularly affecting the cultivation of these small-scale gardens. Residents expressed grievances over inadequate harvests for their daily needs, necessitating the purchase of coconuts and vegetables from the markets. This burden disproportionately affects low-income households, exacerbating economic hardships due to crop losses. Primarily targeted crops, including coconuts, bananas, and vegetables, are staple food items for these communities. Macaques, identified as the primary culprits, consistently damage coconut trees across all study locations, causing major losses by dropping young coconuts to the ground and consuming the soft flesh of mature ones. This destructive behavior results in a diminished overall harvest, evident from the accumulation of immature nuts beside garden plots during field visits. Moreover, macaque incursions occur indiscriminately throughout the day, prolonging the duration and scale of damage. Consequently, some people have abandoned coconut cultivation altogether, deeming it futile and costly. These residents now rely on purchasing coconuts from local markets, thereby foregoing an essential source of supplementary income.

Like elsewhere, the primary target of macaques in home gardens, besides coconuts, was bananas [1,3,6,10]. Macaques not only consume banana fruits but also inflict damage to the trees themselves, diminishing future yields as well. Their preference lies in ripe yellow bananas, though they also feed on a variety of other fruits such as jackfruit, mango, and seasonal vegetables and yams. Being omnivorous, macaques have a broad diet, including leaves, bark, flowers, seeds, roots, cereals, insects, invertebrates, eggs, small mammals, birds, and even human-prepared food. Their adaptability to diverse environmental conditions and large group sizes renders them capable of causing more extensive damage compared to other species. A significant proportion (54%) of complaints received by the Wildlife Department were against macaques, with 70% of these complaints related to crop damage [11]. However, the specific primate species responsible for such damage varies across different regions of the country. For instance, in the Mihintale Kaludiyapokuna forest edge farms, Gray Langurs and Toque Macaques were accountable for 78% and 22% of reported crop damages, respectively [14]. Similarly, a study in Polonnaruwa [2] corroborated these findings, indicating Toque macaques and Gray Langurs as the main culprits behind the human–monkey conflict. Other contributing factors to crop damage include the availability of natural foods, crop variety, seasonal variations, and distance from forests [16].

Aside from primates, crop damage in the area is also attributed to two nocturnal mammals, wild boars, and porcupines, primarily affecting vegetable and yam crops. After Toque macaques, wild boars are the next major animal species attributed to crop destruction, followed by porcupines. The pervasive impact of wildlife-induced crop damage has prompted many residents across the three GN divisions to cease home garden cultivation, resulting in decreased harvests and income. Apart from crop devastation, primates, particularly macaques, are responsible for property damage. The macaques have been observed damaging household items such as pots, pans, plates, rice cookers, and furniture. In unguarded moments, they infiltrate homes, steal stored food from cupboards and racks, defecate indoors, and damage roofs. A similar scenario was documented in the Kandy district, where macaques were reported to forcefully snatch food from peoples’ hands, damage roofs, and vandalize infrastructure [13] Managing human–macaque conflict requires a combination of short-term and long-term strategies. In the long term, promoting the use of forest food through community participation and preparing land use plans for settlements and farmlands can ensure sustainable development while preserving habitats. Controlling settlement expansion and implementing proper monitoring with follow-ups by authorities and locals are crucial for maintaining ecological balance. Short-term measures include using electric fences, conservation programs, and public awareness to mitigate immediate issues. Preventing human–macaque contact, avoiding feeding them, planting non-preferred crops near macaque habitats, and guarding agricultural areas further reduce conflicts. An integrated approach with close collaboration between local communities and authorities is vital for the conservation of protected forests and sustainable coexistence.

## 5. Conclusions

Within the timeline and regional limitations of this study, the extent of crop damage inflicted by macaques on commercial farmers in the study area is significant and is likely to escalate without substantial interventions to control macaque populations. While unauthorized efforts to capture and remove macaques have been undertaken, there has been a lack of comprehensive, wide-scale initiatives to manage macaque populations effectively. The economic impacts outlined here represent the upper limit of the negative consequences associated with macaques. It is far more feasible and cost-effective to control the population of a pest species in its early stages than after it has proliferated. However, with their population growth and expanded range, containing or reducing their numbers now requires a significantly greater effort. If left unaddressed, the management challenges will multiply, resulting in immeasurable losses. Removing entire groups should be avoided, but efforts are needed to reduce group size to a range comparable to non-food-provisioned wild populations to protect local population numbers and reduce crop damage.

In conclusion, an integrated management plan involving relevant stakeholders is necessary to address the conflict between humans and macaques due to crop utilization by macaques.

## Figures and Tables

**Table 1:** The sampling methods used to select sample size sizes from respective GNDs.

Sample Category	Sampling Method Used to Select Sample Size	Sample Size of Deegalla GND	Sample Size of Kabalewa GND	Sample Size of Elathalawa GND
Families Cultivating Commercial Crops/Farmers	Included all the farmers	135	120	20
Families Cultivating Home Garden Crops/Villagers	The Krejcie and Morgan Formula (95% confidence level & 0.05% margin of error)	200	230	170

**Table 2:** Economic loss: Vegetables and Fruits.

Crop Name	No. of Houses	Number of Trees Planted in Home Gardens Before the Onset of HMC (per House)	Number of Trees Planted in Home Gardens After HMC (per House)	Average Monthly Loss of Income from HMC (LKR/per Month)
Areca palm	345	5–25	10–12	2500
Sweet potato	109	3–10	3–5	500
Ceylon oak	40	2–4	1–2	350
Brindle berry	178	3–10	3–4	800
Banana	596	2–5	1–2	1200
Dwarf olive	112	6–8	2–3	1000
Mango	687	6–10	2–5	4000
Papaya	744	2–10	3–5	1500
Maize	287	5–6	2–3	600
Passion fruit	368	4–6	2–3	1500
Rambutan	25	1–4	1	350
Wax apple (Jambu)	99	2–5	1–2	300
Lovi fruit	129	3–4	1–2	250
Graviola	62	4–6	1–2	350
Wood apple	165	3–5	1–2	250
June plum	47	3–6	2–3	300
Cashew	105	3–5	2–3	400
Orange	116	4–6	1–2	450
Guava	242	2–6	1–2	300
Sapodilla	202	5–8	3–4	2500
Betel	404	5–10	5–6	700
Bird chili	300	20	10–12	800
Brinjal	229	3–8	2–4	600
Cassava	277	10–25	5–10	1500
Cantaloupe	126	3–6	2–3	580
Snake gourd	250	10–15	4–5	450
Bitter melon	61	10–15	4–5	350
Drumsticks	65	6–8	2–3	550
Cowpeas	185	3–4	3–4	380
Ladies’ fingers	403	20–40	8–10	500
Spinach	250	10–15	5–6	500

**Table 3:** Property damages made by macaques.

Types of Property Damage	Incidence of Damages (%)	Monthly Cost for Repairs or a Replacement (LKR)
Damage to antennas	58.6	1500–2500
Damage to water taps and water sources	72.6	3500–4000
Roof damage	68.2	1000–1500
Damage to garbage bins	63.8	500–1000
Telephone wires/power lines/bulbs	39.3	850–1500
Home and vehicle mirrors	32.2	2500–3500
Clothes (stealing)	33.2	3500–4500
Essential kitchen items (chill bottles, rice pots, etc.)	40.6	1500–2500

**Table 4:** Calculated economic loss during the fruiting season in the three coconut plantations.

GND	Distance from the Forest (m)	Number of Coconuts Destroyed/per Month During the Fruiting Season	Monthly Total Economic Loss During the Fruiting Season (LKR)	Number of Days Macaques Visited the Plantation/per Month During Fruiting Season
Deegalla (2 acres, 120 trees)	30 m	63–99	5940	23
Kabalewa (1.5 acres, 90 trees)	150 m	71–94	5640	18–22
Elathalawa (3 acres, 200 trees)	200 m	12 -20	1200	8–10

**Table 5:** Calculated economic loss during the non-fruiting season in the three coconut plantations.

GND	Distance from the Forest (m)	Number of Coconuts Destroyed/per Month During Non-Fruiting Season	Monthly Economic Loss During the Non-Fruiting Season (LKR)	Number of Days Macaques Visited the Plantation/per Month During Non-Fruiting Season
Deegalla (2 acres, 120 trees)	30 m	105–220	13,200	28
Kabalewa (1.5 acres, 90 trees)	150 m	106–154	9240	22–25
Elathalawa (3 acres, 200 trees)	200 m	36–60	3600	15–17

**Table 6:** Deterrent methods used by locals against macaques.

Mitigation Actions	Percentage of Method Users	Monthly Cost (LKR)
Firecrackers	90	880–1200
Covering crops with nets	23	2000–2500
Applying black oil to fruit tree trunks	36	1100–2500
Using aluminum sheets to wrap around the coconut tree trunks	41	850–5000
Acrylic masks	79	550–680

## Data Availability

The original contributions presented in the study are included in the article, further inquiries can be directed to the corresponding author.

## Abbreviations

GND	Gramasewa Niladari Division
DCS	Department of Census and Statistics
CBSL	Central Bank Sri Lanka
HMC	Human Macaque Conflict
LKR	Sri Lankan Rupee
